# Assessment of Vascular Endothelial Dysfunction in Septic Patients Using Brachial Flow-Mediated Dilation: A Systematic Review and Meta-Analysis

**DOI:** 10.3390/diagnostics15233021

**Published:** 2025-11-27

**Authors:** Lana Kattan, Sara Abulola, Mohamed H. Elsayed, Abderrezzaq Soltani, Mohamed Izham Mohamed Ibrahim, Zaid H. Maayah

**Affiliations:** 1 Department of Pharmaceutical Sciences, College of Pharmacy, QU Health, Qatar University, Doha P.O. Box 2713, Qatar; 2 QU Health, Qatar University, Doha P.O. Box 2713, Qatar; 3 Clinical Pharmacy and Practice Department, College of Pharmacy, QU Health, Qatar University, Doha P.O. Box 2713, Qatar

**Keywords:** sepsis, septic shock, endothelial dysfunction, flow-mediated dilation

## Abstract

**Background/Objective:** Sepsis remains a major cause of morbidity and mortality worldwide, making early risk stratification and prognosis critical. Vascular endothelial dysfunction is a hallmark of sepsis pathogenesis, with evidence suggesting that endothelial injury may occur early, preceding organ failure. Brachial flow-mediated dilation (FMD), a validated noninvasive ultrasound technique measuring endothelium-dependent vasodilation, serves as a surrogate marker of endothelial function, where lower FMD values reflect impaired function. This systematic review and meta-analysis aimed to evaluate the validity and quality of evidence on using FMD to measure vascular endothelial dysfunction in septic patients by comparing FMD (i) between septic patients and non-septic controls and (ii) between sepsis non-survivors and survivors. **Methods:** PubMed, Embase, Scopus, and Web of Science were searched until November 2024 for clinical studies assessing FMD in septic patients. A random-effects model was used for the meta-analysis, and quality of studies was assessed using the Newcastle–Ottawa Scale. **Results:** Eight studies were included, and seven underwent quantitative synthesis (385 septic patients, 106 non-survivors and 217 survivors). Compared with non-septic controls, septic patients demonstrated significantly lower FMD (pooled standardized mean difference (SMD) = −2.1617; 95% CI −3.8349 to −0.4885; *p* = 0.0113; *I*^2^ = 98.2939%). Within the sepsis cohort, non-survivors showed significantly attenuated FMD compared to survivors (pooled SMD = −0.7003; 95% CI −1.1133 to −0.2873; *p* = 0.001; *I*^2^ = 60.5593%). **Conclusions:** FMD shows potential as a surrogate marker of endothelial dysfunction for sepsis risk assessment, as evident by lower FMD in septic patients, particularly non-survivors.

## 1. Introduction

Sepsis is a life-threatening organ dysfunction resulting from a dysregulated host response to infection [1]. It is a major cause of morbidity and mortality globally, with an incidence of 48.9 million cases and 11 million deaths, accounting for nearly one-fifth of deaths worldwide [2]. Sepsis is recognized as a worldwide health threat by the World Health Organization (WHO) [3], and it poses a substantial economic burden on healthcare systems, with an estimated hospital-related cost of 2.65% of the total healthcare budget [4]. In high-income countries, the average hospital-wide cost of sepsis per patient was estimated to exceed US$ 32,000 [5]. Timely management in sepsis is critical, as for every one-hour treatment delay, the risk of mortality increases by 4–9% [6,7,8]. Despite advancements in medical care, there remains a crucial need for earlier diagnostic approaches and improved clinical management strategies for sepsis.

One of the main organs involved in the pathogenesis of the host response in sepsis is the vascular endothelium [9]. Sepsis is associated with severe endothelial cell dysfunction, which causes dysregulation of hemostasis and vascular reactivity, in addition to tissue edema, increased inflammation, and oxidative stress [9,10,11]. Sepsis-induced vascular endothelial dysfunction is also considered a key contributor to the progression of multiple organ dysfunction syndrome (MODS) [9,10] as supported by strong evidence for the role of impaired vascular endothelial function in sepsis and MODS [10,12], which underscores a link between endothelial dysfunction and poor clinical outcomes in septic patients. Thus, assessing endothelial function in patients with sepsis through bedside monitoring may provide a valuable tool for detection and risk stratification by enhancing our understanding of vascular endothelial impairment and its impact on organ damage [1].

Brachial flow-mediated dilation (FMD), also referred to as brachial artery reactivity (BAR), is an ultrasound-guided tool that quantifies endothelium-dependent vasodilation of the brachial artery, and it is widely considered the gold-standard non-invasive method for measuring endothelial function in humans [13,14]. Using an ultrasound, the changes in brachial artery diameter are measured following ischemia, typically induced by inflating a forearm blood pressure cuff to a level above systolic pressure for a typical duration of five minutes [15]. Clinically, FMD is considered a surrogate marker of vascular health and an independent predictor of risk of future cardiovascular events in patients with cardiovascular disease and non-cardiovascular disease [16,17,18], supporting its potential use as a non-invasive tool for bedside prognostic risk stratification, particularly in diseases in which endothelial dysfunction contributes to pathogenesis, like sepsis.

In sepsis, although multiple narrative reviews have discussed the role of sepsis-induced endothelial dysfunction [9,10,19] evidence supporting the utility of FMD specifically for evaluating the impact of sepsis-induced endothelial dysfunction remains limited. The aim of this systematic review and meta-analysis is to synthesize and examine the validity and quality of the existing evidence that discusses the role of FMD in measuring endothelial dysfunction in sepsis.

## 2. Material and Methods

This systematic review and meta-analysis was conducted according to the “Cochrane Handbook for Systematic Reviews of Interventions” [20] and was reported following the “Preferred Reporting Items for Systematic Reviews and Meta-Analyses (PRISMA)” guidelines [21] (Supplementary Table S1).

### 2.1. Search Strategy

A systematic literature search was carried out in PubMed, Embase, Scopus, and Web of Science from inception until 21 November 2024. The reference lists of all included studies were manually screened for any additional relevant articles. The search strategy was framed according to the “Population, Intervention, Comparison, and Outcome (PICO)” question: “In patients with sepsis or septic shock, does sepsis induce vascular endothelial dysfunction that can be measured using FMD?”. We used the term “sepsis” along with its variations for the population, and “flow-mediated dilation” and its variations for the outcome related to endothelial dysfunction (Supplementary Table S2). Synonyms were combined with “OR” within each component, and components were combined with “AND”. The full search strategy for each database is available in Supplementary Table S2.

### 2.2. Selection Criteria

We included peer-reviewed clinical studies that assessed FMD in septic patients and compared the values either with non-septic controls or between sepsis non-survivors and survivors. We did not specify a definition of sepsis during the selection process to avoid excluding potentially relevant studies that used varying diagnostic criteria. Studies were excluded if the population did not consist of septic patients, lacked measurements from non-septic controls or comparisons between non-survivors and survivors. Conference abstracts, case series, reviews, non-English studies, and non-clinical studies, such as animal studies, were excluded.

The studies identified through the search were uploaded to Rayyan platform [22]. Title and abstract screening was conducted independently by two authors (LK and SA), followed by independent full-text screening of articles by the same authors. Conflicts during the selection process were resolved either by mutual agreement or through consultation with a third author (ZM).

### 2.3. Data Collection

Data extraction was conducted independently by two authors (LK and ME) and subsequently reviewed by a third author (SA) using a predefined data extraction tool in “Microsoft Excel”. Data extraction included (1) study characteristics (first author, publication year, country of origin and study design), (2) patient characteristics (age, gender, sample size, severity of sepsis including SOFA score or APACHE II score, use of vasopressors, mean arterial pressure, and length of hospital stay), (3) study outcomes related to FMD, and (4) timeframe since sepsis onset and FMD measurement. We used the definition of sepsis as reported by the included studies, and we reported the detailed FMD measurement protocol followed by each study.

### 2.4. Quality Assessment

The quality assessment of the included studies was performed independently by three authors (LK, ME, and SA). Inter-rater reliability was not formally calculated; however, discrepancies were resolved through discussion or by consultation with a senior author (ZM) when required to ensure consistency. The “Newcastle-Ottawa Quality Assessment Scale” (NOS) was used to assess the quality of the studies [23]. The NOS tool evaluates the quality of non-randomized studies, including case–control and cohort studies, across three main domains: selection, comparability, and exposure (for case–control studies) or outcome (for cohort studies). Within each domain, individual items are rated, and a study can receive a ‘star’ per item if it meets the criteria, with a maximum of nine stars across all domains, divided as up to four stars for selection, up to two stars for comparability, and up to three stars for exposure or outcome. A study was classified as high quality if the total score was (7–9 stars), moderate quality if (4–6 stars), and poor quality if (0–3 stars).

### 2.5. Statistical Analysis

We performed a meta-analysis of the included studies to assess differences in FMD values in septic patients compared to non-septic controls, as well as to evaluate variations in FMD among sepsis non-survivors and survivors, using Jamovi© software version 2.4.11. FMD was reported as the mean percentage change ± standard deviation (SD) in the diameter of the brachial artery during hyperemia relative to the diameter at baseline (pre-cuff inflation). One study by Ravikumar et al. (2023) [24] calculated FMD as a ratio of post- to pre-deflation cross-sectional area rather than the conventional percentage change in artery diameter.

In two studies, Ravikumar et al. (2023) [24] and Wexler et al. (2012) [25], FMD values were reported as median (range); therefore, using the method described by Hozo et al. (2005) [26], the data were converted to mean and standard deviation. A random-effects model was used to calculate the “pooled standardized mean difference” (SMD) with 95% confidence intervals. To account for variations in the measurements of FMD across the studies, SMD was used to standardize the results on a common scale to estimate the effect size for each study. Results were considered statistically significant if *p* < 0.05. The degree of heterogeneity was evaluated using the Chi-square test and quantified by the *I*^2^ statistic, with heterogeneity considered substantial if *I*^2^ ≥ 50% [20]. Because each meta-analysis included fewer than 10 studies, we could not assess publication bias using funnel plots or Egger’s regression, in line with Cochrane Handbook guidance that the power of these tests is low with small numbers of studies.

## 3. Results

### 3.1. Search Results

**Figure 1:**
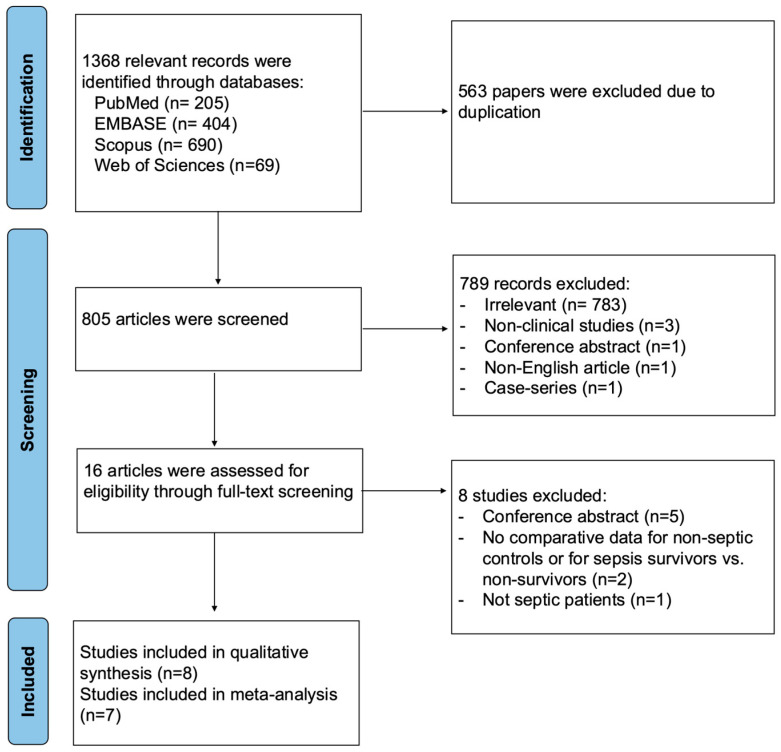
PRISMA flow diagram for studies evaluating endothelial function by FMD in septic patients compared to non-septic controls or between sepsis survivors and non-survivors.

A total of 1368 relevant records were identified through database searches, with 563 duplicates removed. After title and abstract screening, 789 articles were excluded. Eight articles were excluded during full-text screening, and a total of eight studies were included in the qualitative synthesis. One study, Fayed et al. (2022) [27], was excluded from the quantitative synthesis because the data were reported as an interquartile range, which could not be reliably converted to mean and standard deviation (Figure 1).

### 3.2. Study and Patient Characteristics

Out of the eight included studies, four were prospective observational studies [24,28,29,30], three were cross-sectional studies [27,31,32], and one was a combined case–control and prospective cohort study [25]. Studies were published between 2008 and 2023 from South America (Brazil, *n* = 2), North Africa (Egypt, *n* = 2), South Asia (India, *n* = 1), North America (United States, *n* = 2), and Europe (Italy, *n* = 1). A summary of study characteristics is presented in Table 1.

Overall, the studies included 604 septic patients and 449 non-septic controls were included in the studies, all of whom were adults. The reported mean ages of septic patients ranged from 30 to 62 years (Table 1). The proportion of male septic patients ranged from 38% to 65% across the studies, with only two studies reporting a female-dominant cohort [29,30] (Table 1). Among the studies that compared sepsis non-survivors with survivors (*n* = 542 patients), 203 were non-survivors, while 339 were survivors. The mean age ranged from 55 to 71 years in the non-survivors’ group and from 41 to 58 years in the survivors’ group (Table 1). Sex distribution by survival status was reported in four studies [25,28,29,31], with a lower proportion of males among non-survivors (30% to 51%) compared to survivors (39% to 71%) (Table 1).

Pulmonary, intra-abdominal, and urinary infections were the most commonly reported sources of sepsis [24,25,28,29,32]. The majority of patients were admitted to intensive care units (ICUs) [25,27,28,29,31,32] secondary to sepsis or severe sepsis diagnosis; however, only one study excluded patients with organ dysfunction [30]. In addition, the length of ICU stay ranged from 6 to 12 days (Table 1). The timeframe for measuring FMD was similar across the studies, with FMD being assessed within 24 h of ICU admission (Table 2 and Table 3), except one study, where FMD was measured at an average of 41 h after meeting sepsis criteria [25] (Table 2 and Table 3).

Sepsis definitions varied across the included studies, with only one study explicitly using Sepsis-3 diagnostic criteria [31]. Most studies applied SIRS-based Sepsis-1/2 diagnostic criteria [25,28,29,30,32]. However, two studies did not specify a formal sepsis criterion; for example, Ravikumar et al., 2023 included patients with a sepsis model of perforation peritonitis undergoing emergency laparotomy [24], whereas Fayed et al., 2022 included an ICU cohort with sepsis-associated AKI [27].

Sepsis severity was reported using the “Sequential Organ Failure Assessment” (SOFA) and the “Acute Physiology and Chronic Health Evaluation II” (APACHE II) scoring systems. SOFA scores were expectedly higher among non-survivors, ranging from 4 to 10.36, compared to 0.5 to 6.5 in survivors (Table 1). Similarly, APACHE II scores ranged from 14.5 to 28.7 in non-survivors and from 6.5 to 22 in survivors (Table 1). The mean arterial pressure (MAP) was reported in three studies [25,31,32], ranging from 71 mmHg to 94.3 mmHg (Table 1), with a lower MAP in non-survivors compared to survivors (82 mmHg vs. 94.3 mmHg) (Table 1). In addition, 21.6% to 79% of patients received vasopressors across three studies [25,28,29]. Notably, control groups varied across the studies, with four studies including healthy participants [27,29,30,32], two studies including participants without acute illness [25,31], and one study including hemodynamically stable elective-surgery patients [24]. The main patient characteristics in the included studies are summarized in Table 1.

There was notable variability in the protocols used to measure FMD in the included studies. While most studies performed measurements in the supine position with the arm extended and utilized forearm cuff placement inflated to suprasystolic pressures typically between 200 and 259 mmHg for a duration of 4–5 min, differences were observed in the exact imaging site, occlusion duration, probe frequency, and post-deflation acquisition intervals. A summary of the FMD measurement protocols followed in the included studies is presented in Supplementary Table S3.

### 3.3. Quality Assessment of Studies

**Figure 2:**
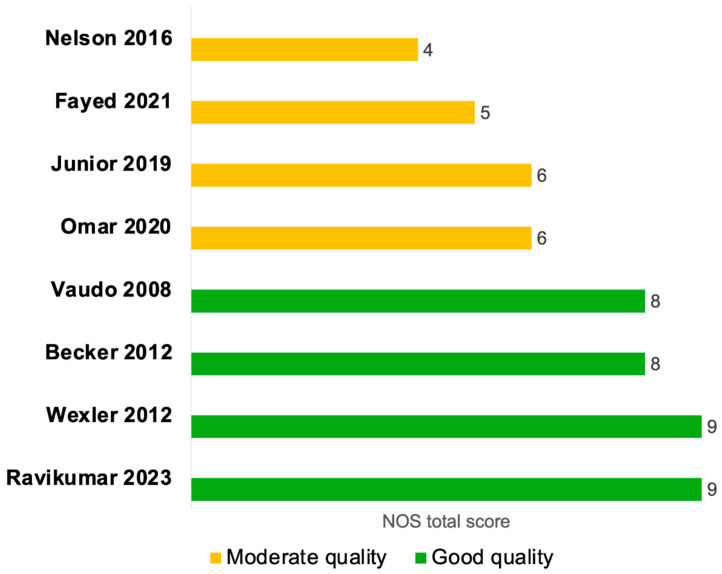
Quality assessment of studies evaluating endothelial function by FMD septic patients compared to non-septic controls or between sepsis survivors and non-survivors using the Newcastle-Ottawa Quality Assessment Scale (NOS). The NOS tool evaluates the quality of case–control and cohort studies across three main domains: Selection, Comparability, and Exposure (for case–control studies) or Outcome (for cohort studies). Within each domain, individual items are rated, and a study can receive a ‘star’ for each item if it meets the criteria, with a maximum of nine stars across all domains, divided as up to four stars for selection, up to two stars for comparability, and up to three stars for exposure or outcome. A study was classified as high quality if the total NOS score was (7–9 stars), moderate quality if (4–6 stars), and poor quality if (0–3 stars) [24,25,27,28,29,30,31,32].

Six studies were evaluated using the NOS cohort checklist [24,25,28,29,30,31], and two studies were assessed by the NOS case–control checklist [27,32] (Figure 2 and Supplementary Table S4). Four of the cohort studies were of high quality [24,25,29,30], with clearly defined and well-reported cohort selection, appropriate comparability across cohorts in terms of confounders, and valid outcome assessment (Figure 2 and Supplementary Table S4A). Two cohort studies were of moderate quality [28,31], primarily due to insufficient adjustment for confounding variables in the comparability domain. Moreover, the study by Junior et al., 2019 [28] was a single-arm cohort study; therefore, the NOS item assessing the selection of a non-exposed cohort was not applicable and was assigned a score of zero (Figure 2 and Supplementary Table S4A). The two case–control studies were of moderate quality, mainly because the two NOS items in the exposure domain were inherently not applicable to their study designs and were scored as zero, since the presence of sepsis itself was considered as the exposure. Additionally, the non-response rate was not clearly reported in these studies (Figure 2 and Supplementary Table S4B).

### 3.4. FMD in Septic Patients Compared to Non-Septic Controls

**Figure 3:**
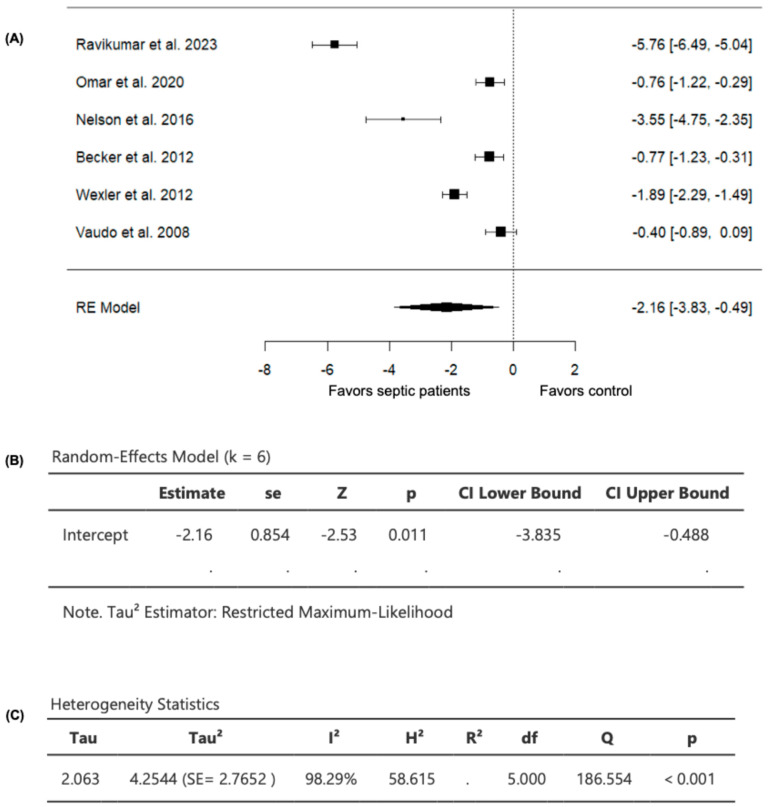
FMD in septic patients and non-septic controls. (**A**) Forest plot of standardized mean differences (SMD). (**B**) Random-effects model summary. (**C**) Heterogeneity statistics.

Six studies comparing FMD values in septic patients (*n* = 385) and non-septic controls (*n* = 230) were included in the analysis (Table 2). The observed SMD ranged from −5.7614 to −0.3997, with all studies demonstrating lower FMD values in septic patients compared to non-septic controls (Figure 3), suggesting consistently worsened vascular endothelial function in patients with sepsis compared to controls. For the pooled FMD data, the average estimated SMD was −2.1617 (95% CI: −3.8349 to −0.4885) among patients with sepsis, as determined by the random-effects model (Figure 3). As a result, the average outcome showed a significant difference from zero (z = −2.5322, *p* = 0.0113), supporting the impact of sepsis on FMD values (Figure 3).

A substantial level of heterogeneity was observed across the studies as indicated by the Q test (Q = 186.5536, *p* < 0.0001, *I*^2^ = 98.2939%) (Figure 3). In addition, analysis of the studentized residuals revealed that one study (Ravikumar et al., 2023 [24]) was identified as a potential outlier in this model, with a value greater than ± 2.6383. Furthermore, based on Cook’s distances, (Ravikumar et al., 2023 [24]) was also considered to be overly influential.

**Figure 4:**
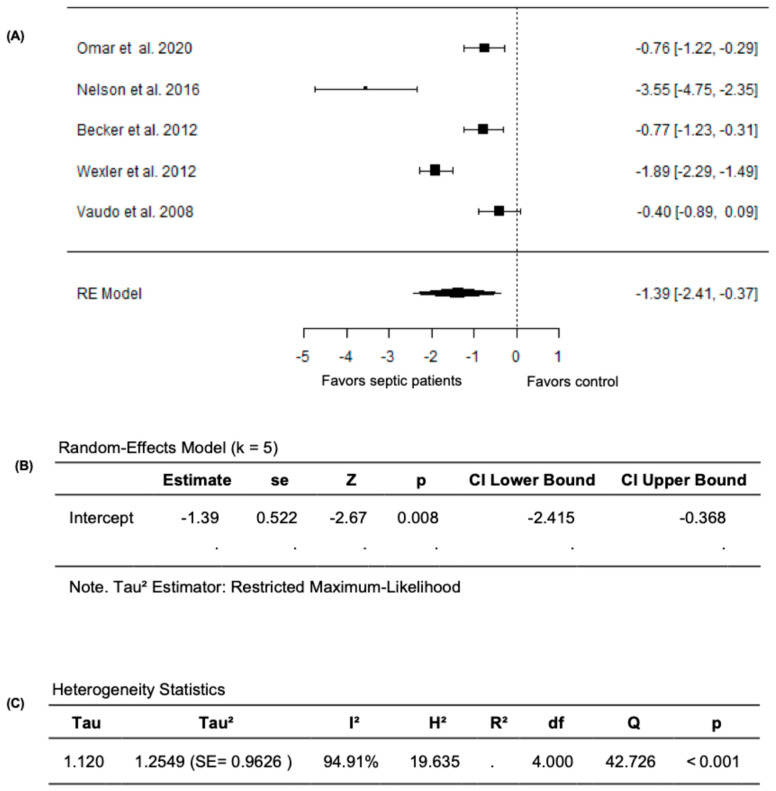
FMD in septic patients and non-septic controls after excluding Ravikumar et al. (2023) [24]. (**A**) Forest plot of standardized mean differences (SMD). (**B**) Random-effects model summary. (**C**) Heterogeneity statistics.

Given that the study by Ravikumar et al. (2023) [24] measured FMD as the change in the cross-sectional area instead of the conventional percentage change in brachial artery diameter, we performed a meta-analysis excluding this study. In this analysis, the pooled FMD remained significantly lower in septic patients compared to non-septic controls after excluding the study by Ravikumar et al. (2023) [24]. The random-effects model yielded a pooled SMD of −1.3913 (95% CI: −2.4145 to −0.3681) and the average outcome remained significantly different from zero (z = −2.6650, *p* = 0.0077) (Figure 4A,B). However, heterogeneity remained substantial across the studies as indicated by the Q test (Q = 42.7264, *p* < 0.0001, *I*^2^ = 94.9071%) (Figure 4C). Following the evaluation of the studentized residuals, one study, Nelson et al. (2016) [32], was identified as a potential outlier in this model with a value greater than ±2.5758; however, none of the studies were found to be overly influential as per the Cook’s distances. Overall, these findings establish the impact of sepsis on vascular endothelial function, with markedly lower FMD values in septic patients compared to non-septic controls.

### 3.5. FMD in Sepsis Non-Survivors Compared to Survivors

**Figure 5:**
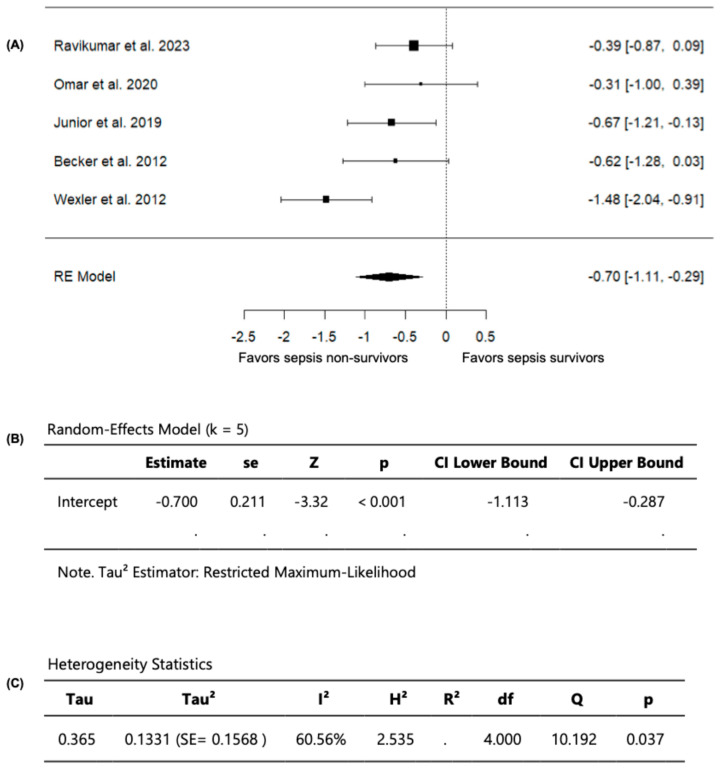
FMD in sepsis non-survivors and survivors. (**A**) Forest plot of standardized mean differences (SMD). (**B**) Random-effects model summary. (**C**) Heterogeneity statistics.

Out of the seven included studies, five studies, with a total of 323 septic patients (106 non-survivors and 217 survivors) compared FMD values among sepsis non-survivors and survivors and were included in the analysis (Table 3). In these studies, the observed SMD ranged from −1.4784 to −0.3055 (Figure 5). Moreover, across all studies, FMD values were persistently lower in sepsis non-survivors compared to survivors, indicating that impaired vascular endothelial function is associated with worse outcomes in sepsis, including mortality. Using a random-effects model, the pooled SMD was −0.7003 (95% CI: −1.1133 to −0.2873) (Figure 5). Accordingly, the average outcome demonstrated a significant difference from zero (z = −3.3232, *p* = 0.0009), confirming that sepsis non-survivors have lower FMD values than survivors (Figure 5).

Based on the Q-test, substantial heterogeneity was observed in the outcome of measuring FMD across the studies (Q = 10.1923, *p* = 0.0373, *I*^2^ = 60.5593%)**.** Upon examining the studentized residuals, one study (Wexler et al., 2012 [25]) had a value greater than ±2.5758 and was considered a potential outlier in this model. Following the evaluation of Cook’s distances, no study was overly influential. Collectively, these findings confirm that sepsis non-survivors have greater vascular endothelial dysfunction, evident with lower FMD values compared to survivors.

**Figure 6:**
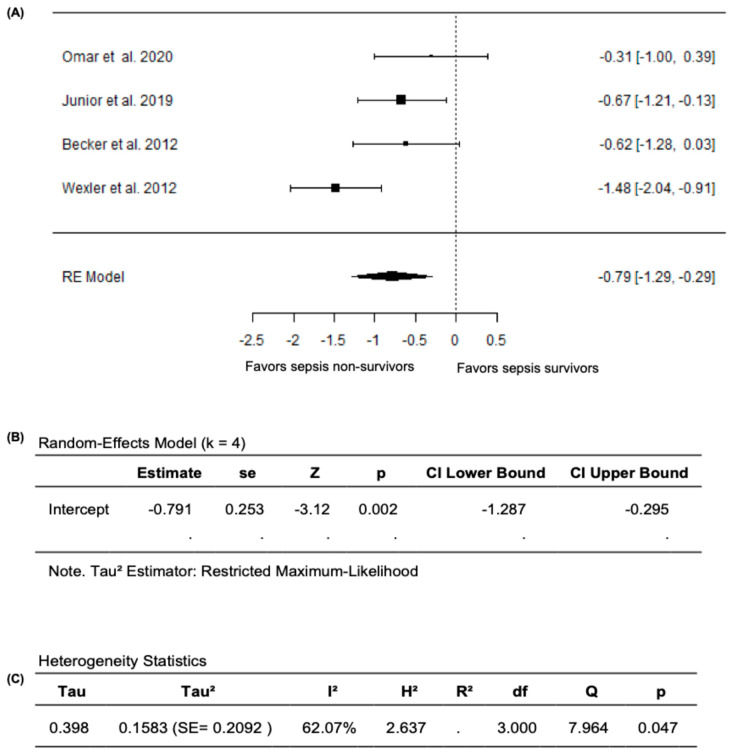
FMD in sepsis non-survivors and survivors after excluding Ravikumar et al. (2023) [24]. (**A**) Forest plot of standardized mean differences (SMD). (**B**) Random-effects model summary. (**C**) Heterogeneity statistics.

In the meta-analysis that excluded Ravikumar et al. (2023) [24], based on the random effects model, the new pooled SMD was −0.7906 (95% CI: −1.2866 to −0.2945), with the average outcome exhibiting a significant difference from zero (z = −3.1236, *p* = 0.0018), which supports the validity of the main findings of reduced FMD values in sepsis non-survivors compared survivors (Figure 6A,B). Nevertheless, substantial heterogeneity remained across the studies according to the Q-test (Q= 7.9644, *p* = 0.0468, *I*^2^ = 62.0748%) (Figure 6C). After analyzing the studentized residuals, one study by Wexler et al. (2012) [25] had a value greater than ±2.4977 and was considered a potential outlier in this model. Based on the analysis of Cook’s distances, no study was considered overly influential. Overall, when the analyses were restricted to studies that only reported FMD as the percentage change in brachial-artery diameter during hyperemia relative to baseline, FMD values remained significantly lower both in septic patients compared to non-septic controls and in non-survivors compared to survivors, confirming the robustness of our primary findings.

## 4. Discussion

Sepsis continues to be a major health burden that is associated with high mortality and morbidity [33]. Early sepsis recognition and timely management are essential for improved outcomes [6,7,8,34]. In addition, effective risk stratification and prognosis are critical, as identifying high-risk patients may prompt earlier, more aggressive management while sparing low-risk patients from unnecessary interventions [35]. Given the key role of the vascular endothelium in the pathogenesis of sepsis [9], along with evidence suggesting that endothelial injury may be an early process preceding organ failure [36], the use of a reliable non-invasive bedside method that can evaluate endothelial dysfunction could potentially facilitate early risk stratification and prognosis in sepsis. This would allow tailored management and monitoring, ultimately improving patient and clinical outcomes.

Endothelial dysfunction is characterized by a decrease in the production of or sensitivity to nitric oxide (NO), a potent vasodilator that regulates vascular homeostasis [37]. FMD reflects the endothelium-dependent vasodilatory response of a vessel to shear stress, which triggers the release of NO [38]. Therefore, FMD can be considered as an indicator of NO release, where lower FMD values reflect less vasodilation, lower NO activity, and, thus, endothelial dysfunction. FMD is the gold-standard non-invasive method for measuring endothelial function, which has been widely validated and closely associated with coronary artery function [14]. The role of FMD in evaluating endothelial dysfunction has been established in various disease states [39,40,41]. However, the validity and quality of the current literature evaluating the use of FMD as a method for measuring the impact of sepsis on vascular endothelial dysfunction remain unclear. To our knowledge, this is the first systematic review and meta-analysis to assess the validity of clinical studies using FMD as a measure of vascular endothelial dysfunction in septic patients, including both survivors and non-survivors.

Our systematic review and meta-analysis demonstrated that septic patients consistently had significantly lower FMD values compared to non-septic controls, with sepsis non-survivors showing even greater impairment compared to survivors. Nevertheless, the magnitude of FMD reduction varied across the studies. Notably, the study by Ravikumar et al., 2023 [24] reported the lowest FMD values among all groups, including septic patients and non-septic controls, as well as sepsis non-survivors and survivors. This discrepancy may be attributed to methodological differences, as Ravikumar et al. (2023) [24] reported FMD as a ratio of the change in the cross-sectional area rather than the percentage change in arterial diameter as reported by all other studies. Given that area scales with the square of diameter, equivalent biological changes can appear numerically larger when expressed this way. Importantly, in the meta-analysis excluding this study, our findings remained statistically significant, with lower FMD values in septic patients compared to non-septic controls and in sepsis non-survivors compared to survivors. Nevertheless, this exclusion did not eliminate between-study heterogeneity; therefore, the pooled estimates should be interpreted with caution. Conversely, the study by Vaudo et al. (2008) [30] reported markedly higher FMD values, approximately 2- to 9-fold higher than those in the remaining studies [24,25,29,31,32] for both septic patients and their non-septic controls. This could be explained by the fact that the study was restricted to patients with Gram-negative sepsis and explicitly excluded patients with organ dysfunction. Thus, the cohort could potentially have a milder clinical phenotype with preserved endothelial function. However, given that the non-septic controls in this study have also demonstrated higher FMD values relative to other studies, the difference cannot be explained by sepsis severity alone and is likely due to other additional factors, such as methodological differences, particularly in the cuff inflation protocol, as well as the overall younger age of the study population compared to the cohorts in the other studies.

Impairment of the vascular endothelium is a common hallmark of various acute inflammatory conditions such as burns, trauma, and acute respiratory distress syndrome (ARDS), including that caused by COVID-19, as well as sepsis [42]. Our findings align with a recent meta-analysis demonstrating that individuals recovering from COVID-19 showed reduced FMD compared to controls [43]. In sepsis, endothelial dysfunction emerges from different pathophysiological mechanisms, including inflammation, oxidative stress, and glycocalyx shedding, which results in an increase in vascular leakage, impairment of microcirculation, activation of coagulation pathways, and worsening of multiple organ dysfunction [10,44]. A key driver is the cytokine storm, triggered by a pathogen-induced inflammatory cascade during severe inflammation [44]. This endothelial inflammatory response in sepsis contributes to enhanced oxidative damage from reactive oxides such as NO and reactive oxygen species (ROS), which cause endothelial dysfunction by impairing the mitochondrial function of endothelial cells, reducing antioxidant capacity, and inducing apoptosis [10]. Moreover, inflammatory response modulated by the endothelium involves the activation of endothelial NF-κB and NLRP3 pathways, which in turn enhances the expression of different pro-inflammatory cytokines such as TNF-α and IL-6. As a response, this also increases the expression of cell-adhesion molecules, such as intercellular adhesion molecule-1 (ICAM-1) and vascular cell adhesion molecule-1 (VCAM-1), where the levels of cell-adhesion molecules directly correlate with sepsis severity and organ dysfunction [44]. Consistent with this, Becker et al. (2012) [29] reported a significant negative correlation between FMD and both IL-6 and sVCAM-1, suggesting that FMD may also reflect broader inflammatory and vascular activation signals. This supports the validity of FMD as a biologically meaningful and clinically relevant marker of endothelial dysfunction in sepsis.

The clinical relevance of FMD is further supported by its correlations with validated sepsis severity scores and metabolic markers across several studies in our review. For instance, FMD showed a significant negative correlation with the APACHE II score [32] and changes in the SOFA score [30]. This suggests that vascular impairment, as measured by FMD, is associated with the severity of sepsis, indicating that FMD may also serve as a surrogate marker of a wider range of sepsis-induced inflammatory dysregulation. Notably, Vaudo et al. (2008) [30] observed that a rise in FMD during the first 72 h of sepsis diagnosis was associated with a decrease in SOFA, suggesting that dynamic FMD monitoring may identify patients who are recovering vascular homeostasis, which could complement other conventional sepsis severity risk scores. Furthermore, FMD was significantly correlated with metabolic parameters, including lactate [29] and lactate clearance [24], both of which are strongly associated with an increased risk of mortality in sepsis [45]. Taken together, these associations support the utility of FMD as an integrative bedside tool for sepsis prognosis.

Notably, it is essential to highlight the potential confounding effect of vasopressors, which are integral in the management of septic shock to maintain adequate perfusion and mean arterial pressure [46]. Vasopressors might have a significant impact on endothelial function [47]. Catecholamines, particularly norepinephrine and epinephrine, were found to reduce Toll-like receptor-mediated endothelial permeability, suggesting a direct effect on endothelial barrier function [47]. Furthermore, excessive administration of catecholamines may further exacerbate glycocalyx damage, one of the key mechanisms of endothelial impairment in sepsis [48]. Traditionally, FMD guidelines recommend that all vasoactive medications be withheld for at least four half-lives before measurement [49]. However, in a clinical study of healthy volunteers, it has been shown that the administration of non-nitrate-containing vasoactive medications did not impact FMD, suggesting that the practice of withholding these medications may not be necessary [50]. In the present systematic review, we observed a variation in the reporting of vasopressor use among the included studies, which may be a potential confounder when interpreting FMD in critically ill patients.

In sepsis ICU cohorts in the included studies, brachial-artery FMD was feasible at the bedside using portable ultrasound with the patient in a supine position (forearm cuff, suprasystolic inflation, ~5 min occlusion), supporting its use in critically ill adults. However, there might be some challenges for utilizing FMD in the ICU, such as quantifying the effect of vasoactive drugs on FMD evaluation [32], as well as the potential influences of sedatives and mechanical ventilation [28]. Moreover, in some instances, when vascular-access devices are present in the target upper extremity, FMD measurements may be delayed, which may prevent earlier or serial measurements [25].

Overall, the significant decrease in FMD among septic patients, particularly non-survivors, underscores the need for routine bedside, non-invasive assessment of endothelial function. Considering that endothelial impairment may precede organ dysfunction, FMD can be a valuable early marker for identifying patients at risk of developing MODS and having worse outcomes. Nevertheless, our study has some limitations that warrant discussion. First, only English-language studies were included, which may have introduced publication bias, and both the number of included studies and the total sample size of patients were limited, which may limit the generalizability of the findings. In addition, the potential impact of vasopressor use on FMD was not quantitatively assessed. Moreover, the observational nature of the included studies precludes causal inference and should be viewed only as a hypothesis-generating study. Furthermore, substantial heterogeneity was present across studies, which we attempted to address using a random-effects model in the analysis.

Heterogeneity is another important limitation and may be attributed to various factors; first, there were variations in sepsis definitions used across the studies, as sepsis diagnostic criteria have changed considerably over time [51]. This involved moving from SIRS-based definitions, as used in Sepsis-1 and Sepsis-2 criteria [52,53], to SOFA-based definitions as used in Sepsis-3 [1]. It is important to note that Sepsis-3 criteria define sepsis as a life-threatening organ dysfunction caused by a dysregulated host response to infection, which inherently incorporates “severe sepsis” into the definition, and define septic shock more stringently as vasopressors required to keep MAP ≥ 65 mmHg and serum lactate >2 mmol/L despite adequate fluid resuscitation [1]. This suggests the need for explicit, consistent reporting of Sepsis-3 criteria in future studies. Other factors which may have contributed to heterogeneity among studies include variability in FMD measurement protocol, measurement conditions, scanning techniques, and analysis. This emphasizes the need for standardized FMD assessment protocols. Also, the lack of a universally accepted diagnostic threshold for FMD limits its integration into routine clinical practice. While Junior et al. (2019) suggested that septic patients whose baseline FMD% was above −1% had a prolonged survival and lower risk of death at 28 days post-diagnosis [28], this FMD cut-off point for mortality prediction in sepsis still requires further validation in other cohorts. Therefore, multicenter randomized controlled trials are needed to generate robust evidence on the clinical value of FMD in evaluating sepsis-related endothelial dysfunction and to clarify its potential role in prognosis and early risk stratification. Lastly, since we lacked access to raw patient data, we could not perform a meta-regression to examine whether the interval between sepsis onset and FMD measurement or the use of vasoactive agents affected the results.

## 5. Conclusions

Our study demonstrates that brachial FMD, a surrogate marker of endothelial function, is significantly lower in adult patients with sepsis compared to non-septic controls. In addition, the results suggest that FMD is associated with sepsis survival, with non-survivors showing lower FMD values compared to survivors. However, these results should be interpreted with caution given the small sample size, the substantial heterogeneity among the studies, the observational nature of the included studies, and the absence of randomized controlled trials. In aggregate, our study provides proof-of-concept evidence that supports the potential role of FMD monitoring as a promising non-invasive bedside technique for detecting vascular endothelial dysfunction in septic patients, which could aid in risk stratification and prognosis. Future studies should define and validate a standardized FMD cut-off value for predicting sepsis outcomes. Given the central role of vascular endothelium in sepsis pathogenesis and progression, implementing clinical interventions aimed at preserving or restoring endothelial integrity in sepsis may help limit progression to multiple-organ dysfunction and ultimately improve sepsis survival.

## Figures and Tables

**Table 1:** Characteristics of clinical studies assessing flow-mediated dilation in septic patients.

Study	Country	Study Design	Study Population	Age, Years ^a^	Sex	Sepsis Severity ^b^	Blood Pressure, mmHg ^c^	LOS, Days ^d^	Outcomes
Ravikumar et al., 2023 [24]	India	Prospective cohort study	Adult patients with perforation peritonitis undergoing emergency laparotomy (*n* = 76), including survivors (*n* = 50) and non-survivors (*n* = 26), and their controls of adult patients undergoing elective laparotomy without any features of sepsis (*n* = 75).	30 (23–45) for patients with perforation peritonitis undergoing emergency laparotomy. 42 (30–52) for control patients undergoing elective laparotomy.	65% males for patients with perforation peritonitis undergoing emergency laparotomy	SOFA score: 2 (0–4) overall; 0.5 (0–2) for survivors; 4 (2–6) for non-survivors. APACHE II score: 11 (4–15.5) for all patients. 6.5 (414) for survivors. 14.5 (11–20) for non-survivors.	NR	Length of hospital stay: 9.5 (5–28) for all patients; 15.5 (6–29) for survivors and 6 (2–19) for non-survivors	Significantly lower FMD in sepsis patients compared to controls before laparotomy, immediately after, and 48 h after laparotomy. No significant difference in FMD between survivors and non-survivors. Postoperative FMD was not a good predictor of in-hospital mortality. Significant positive correlation between FMD and lactate clearance at 24 h post-surgery
Fayed et al., 2022 [27]	Egypt	Cross- sectional case–control study	Patients in the ICU with AKI as a consequence of severe sepsis (*n* = 219), including survivors (*n* = 122) and non-survivors (*n* = 97), and their age-matched healthy controls (*n* = 219)	57 (27) for patients with sepsis-induced AKI. 56 (13) for healthy controls	NR	Severe sepsis: all patients had organ dysfunction, evident as acute kidney injury	NR	NR	Significantly reduced FMD in patients with AKI as a consequence of severe sepsis compared to healthy controls. No significant difference in FMD between survivors and non-survivors.
Omar et al., 2020 [31]	Egypt	Cross- sectional study	Adult patients within 48 h of sepsis diagnosis, including those with pulmonary (*n* = 28), abdominal (*n* = 8), UTI (*n* = 25), skin/catheter site (*n* = 14) as sources of sepsis, admitted to the ICU (*n* = 50), including survivors (*n* = 40) and non-survivors (*n* = 10) and their controls without acute illness (*n* = 30).	61.24 ± 13.81 for patients with sepsis. 58.78 ± 13.53 for survivors and 71.10 ± 10.55 for non-survivors. 43.73 ± 16.12 for controls.	50% males in the survivors group. 30% males in the non-survivors.	SOFA score:1.91 ± 2.15 for survivors.10.36 ± 3.34 for non-survivors.	MAP: 94.29 ± 11.94 for survivors. 82.50 ± 8.89 for non-survivors.	NR	Significantly impaired FMD in sepsis patients compared to healthy controls. No significant difference in FMD between sepsis survivors and non-survivors FMD was not a predictor of hospital mortality in the sepsis group
Junior et al., 2019 [28]	Brazil	Prospective cohort study	Adult patients on mechanical ventilation admitted to the ICU with a diagnosis of sepsis (*n* = 60) from respiratory tract (*n* = 30), intra-abdominal (*n* = 27), and other sources (*n* = 3), including survivors (*n* = 21) and non-survivors (*n* = 39)	41.2 ± 14.9 for survivors. 55.2 ± 11.1 for non-survivors.	58.3% males for sepsis patients. 71% males for survivors. 51% males for non-survivors.	SOFA score: 6.5 ± 4 for survivors. 10.3 ± 3.1 for non-survivors. APACHE II score: 21.9 ± 10.3 for survivors. 28.7 ± 6.1 for non-survivors. 63% of patients were using noradrenaline (*n* = 38); 21.6% of patients were using dobutamine (*n* = 13).	NR	Time in ICU: 9.6 ± 6.8 for survivors. 12.3 ± 9.6 for non-survivors.	Significantly lower FMD in sepsis non-survivors compared to non-survivors. FMD values greater than the cutoff of −1% were associated with longer survival and a lower 28-day mortality risk.
Nelson et al., 2016 [32]	United States	Cross-sectional study	Patients with severe sepsis (*n* = 14) or septic shock (*n* = 3) within 48 h of admission to the medical ICU (*n* = 17), and their healthy age- and sex-matched controls (*n* = 16), where FMD was measured in only 11 controls	59 ± 14 for sepsis group. 59 ± 15 for controls	59% male for sepsis group. 56% males for controls	SOFA score: 6 ± 3 APACHE II score: 17 ± 7	MAP: 71 ± 18 for sepsis patients.	Average length of stay in the ICU was ~3	Significantly reduced FMD in septic patients compared to healthy controls. Significant inverse correlation between FMD and APACHE II score.
Becker et al., 2012 [29]	Brazil	Prospective cohort study	Adults within 24 h of diagnosis of severe sepsis or septic shock (*n* = 42), from abdominal (*n* = 19), respiratory (*n* = 12), urinary (*n* = 6), or other source of infection(*n* = 5), including survivors (*n* = 28) and non-survivors (*n* = 14), and their healthy age- and sex-matched controls (*n* = 38) where FMD was measured in only 37 controls	51 ± 19 for sepsis patients. 48 ± 20 for survivors. 57 ± 15 for non-survivors. 47 ± 14 for controls.	38% males for sepsis group. 39% males for survivors. 36% males for non-survivors. 43% males for controls.	APACHE II score: 23 ± 7 overall; 22 ± 6 for survivors and 25 ± 8 for non-survivors. 79% of patients (*n* = 33) required vasopressors.	NR	ICU stay: 8 ± 7	Significantly lower FMD in septic patients compared to healthy controls. Significantly lower FMD in non-survivors compared to survivors at 72 h after sepsis onset Significant negative correlations between FMD and lactate levels, IL-6 and sVCAM-1.
Wexler et al., 2012 [25]	United States	Combined case–control and prospective cohort study	Patients with severe sepsis or septic shock from pulmonary (60%), intra-abdominal (12%), urinary (12%), skin/catheter (4%), or other sources (13%), admitted to the medical and surgical ICU (*n* = 95), including survivors (*n* = 78) and non-survivors (*n* = 17), and their controls without acute illness (*n* = 52)	62 (49–74) for severe sepsis patients. 60 (53–66) for controls	52% males for severe sepsis patients. 55% males for survivors. 35% males for non-survivors. 50% males for controls	APACHE II score: 23 ± 8 85% of patients had severe sepsis (*n* = 73). 83% of survivors had septic shock. 94% of non-survivors had septic shock. 28% of sepsis patients required vasopressors. All patients had at least one dysfunctional organ	MAP: 80 (72–90)	NR	Significantly lower FMD in severe sepsis patients compared to controls, but no significant difference in FMD between severe sepsis survivors and non-survivors. No significant correlation between FMD and SOFA score, number of organ failure-free days, ICU-free days, or ventilator-free days.
Vaudo et al., 2008 [30]	Italy	Prospective cohort study	Patients with Gram-negative sepsis without organ dysfunction (*n* = 45) and their healthy age and sex matched controls (*n* = 25)	41 ± 8 for sepsis patients. 43 ± 5 for controls.	40% males for sepsis patients. 44% males for controls.	SOFA score: 4 ± 1. Patients with organ dysfunction were excluded.	SBP:118 ± 13; DBP: 69 ± 6	NR	Significantly impaired FMD in patients with Gram-negative sepsis compared to controls. Significant positive correlation between brachial FMD, white blood cell count, and changes in SOFA score.

^a^ Age is reported as mean ± SD or median (IQR). ^b^ Sepsis severity was reported based on the definitions or scoring systems used in each study, including SOFA, APACHE II, clinical classifications (e.g., severe sepsis, septic shock), and vasopressor use. SOFA and APACHE II scores are presented as either mean ± or median (IQR). ^c^ Studies reported either mean arterial pressure (MAP) or systolic/diastolic blood pressure (SBP/DBP), as available. Values are presented as mean ± SD or median (IQR). ^d^ Length of stay is presented as either mean ± or median (IQR). SOFA: Sequential Organ Failure Assessment; APACHE II: Acute Physiology and Chronic Health Evaluation II; FMD: Flow-Mediated Dilation; ICU: Intensive care unit; NR: Not Reported; MAP: Mean Arterial Pressure; UTI: Urinary tract infection, sVCAM-1: soluble vascular cell adhesion molecule-1; IL-6: interleukin-6; BP: blood pressure; SBP: systolic blood pressure; DBP: diastolic blood pressure.

**Table 2:** FMD values of sepsis patients and non-septic controls in clinical studies.

Study	Non-Septic Controls FMD (%) Mean ± SD	Sepsis Patients FMD (%) Mean ± SD	Timeframe of Measuring FMD
Ravikumar et al., 2023 [24] *	1.13 ± 0.028 *n* = 75	0.98 ± 0.0233 *n* = 76	Immediately before undergoing laparotomy surgery
Omar et al., 2020 [31]	5.29 ± 1.74 *n* = 30	3.72 ± 2.22 *n* = 50	Within 24 h of ICU admission
Nelson et al., 2016 [32]	6.8 ± 1.3 *n* = 11	1.1 ± 1.7 *n* = 17	25 ± 13 h after ICU admission
Becker et al., 2012 [29]	6 ± 4 *n* = 37	1.5 ± 7 *n* = 42	After 24 h of ICU admission of sepsis patients
Wexler et al., 2012 [25] *	4.11 ± 0.93 *n* = 52	2.65 ± 0.66 *n* = 95	41 (30 to 57) hours after meeting severe sepsis criteria
Vaudo et al., 2008 [30]	9.9 ± 1.1 *n* = 25	8.7 ± 3.6 *n* = 45	At admission to the ICU (within 24 h of diagnosis of sepsis)

* Values originally reported as median (range) were converted to mean ± SD using Hozo et al. [26]’s method. FMD: Flow-mediated dilation; ICU: Intensive care unit; *n*: Number of participants.

**Table 3:** FMD values of sepsis non-survivors and survivors in clinical studies.

Study	Sepsis Non-Survivors FMD (%) Mean ± SD	Sepsis Survivors FMD (%) Mean ± SD	Timeframe of Measuring FMD
Ravikumar et al., 2023 [24] *	1.04 ± 0.03 *n* = 26	1.05 ± 0.0225 *n* = 50	24 h after laparotomy surgery
Omar et al., 2020 [31]	3.17 ± 1.62 *n* = 10	3.86 ± 2.34 *n*= 40	Within 24 h of ICU admission
Junior et al., 2019 [28]	−2.5 ± 15.5 *n* = 39	10.1 ± 23.3 *n* = 21	With the first 24 h after sepsis diagnosis
Becker et al., 2012 [29]	0.8 ± 6 *n* = 14	3.8 ± 4 *n* = 28	24 h after ICU admission
Wexler et al., 2012 [25] *	1.97 ± 0.68 *n*= 17	2.96 ± 0.66 *n* = 78	41 (30 to 57) hours after meeting severe sepsis criteria

* Values originally reported as median (range) were converted to mean ± SD using Hozo et al. [26]’s method. FMD: Flow-mediated dilation; ICU: Intensive care unit; *n*: Number of participants.

## Data Availability

The original contributions presented in the study are included in the article/Supplementary Material, further inquiries can be directed to the corresponding author.

## Supplementary Materials

The following supporting information can be downloaded at: https://www.mdpi.com/article/10.3390/diagnostics15233021/s1, Table S1: PRISMA 2020 Checklist; Table S2: Full Search Strategy; Table S3: FMD ultrasound protocols in the included studies; Table S4: Newcastle-Ottawa scale (NOS) for quality assessment of studies.
