# Supplementary material for: Assessment of Vascular Endothelial Dysfunction in Septic Patients Using Brachial Flow-Mediated Dilation: A Systematic Review and Meta-Analysis
Source: Diagnostics (Basel). 2025 Nov 27;15(23):3021. doi: 10.3390/diagnostics15233021 (PMC12691328; doi:10.3390/diagnostics15233021)
Supplement: Supplementary file 1 [file diagnostics-15-03021-s001.zip › diagnostics-3880714-SI.pdf]

Supplementary table 1. PRISMA 2020 Checklist

Section and Topic	Item #	Checklist item	Location where item is reported
**TITLE**			
Title	1	Identify the report as a systematic review.	p.1
**ABSTRACT**			
Abstract	2	See the PRISMA 2020 for Abstracts checklist.	p.2
**INTRODUCTION**			
Rationale	3	Describe the rationale for the review in the context of existing knowledge.	p. 3,4
Objectives	4	Provide an explicit statement of the objective(s) or question(s) the review addresses.	p.4
**METHODS**			
Eligibility criteria	5	Specify the inclusion and exclusion criteria for the review and how studies were grouped for the syntheses.	p.5
Information sources	6	Specify all databases, registers, websites, organisations, reference lists and other sources searched or consulted to identify studies. Specify the date when each source was last searched or consulted.	p.5
Search strategy	7	Present the full search strategies for all databases, registers and websites, including any filters and limits used.	Supplementary table 1
Selection process	8	Specify the methods used to decide whether a study met the inclusion criteria of the review, including how many reviewers screened each record and each report retrieved, whether they worked independently, and if applicable, details of automation tools used in the process.	p.5
Data collection process	9	Specify the methods used to collect data from reports, including how many reviewers collected data from each report, whether they worked independently, any processes for obtaining or confirming data from study investigators, and if applicable, details of automation tools used in the process.	p.6
Data items	10a	List and define all outcomes for which data were sought. Specify whether all results that were compatible with each outcome domain in each study were sought (e.g. for all measures, time points, analyses), and if not, the methods used to decide which results to collect.	p.6
	10b	List and define all other variables for which data were sought (e.g. participant and intervention characteristics, funding sources). Describe any assumptions made about any missing or unclear information.	p.6
Study risk of bias assessment	11	Specify the methods used to assess risk of bias in the included studies, including details of the tool(s) used, how many reviewers assessed each study and whether they worked independently, and if applicable, details of automation tools used in the process.	p.6, 7
Effect measures	12	Specify for each outcome the effect measure(s) (e.g. risk ratio, mean difference) used in the synthesis or presentation of results.	p.7
Synthesis methods	13a	Describe the processes used to decide which studies were eligible for each synthesis (e.g. tabulating the study intervention characteristics and comparing against the planned groups for each synthesis (item #5)).	p.6
	13b	Describe any methods required to prepare the data for presentation or synthesis, such as handling of missing summary statistics, or data conversions.	p.7
	13c	Describe any methods used to tabulate or visually display results of individual studies and syntheses.	Tables 1–3, Fig. 3-8
	13d	Describe any methods used to synthesize results and provide a rationale for the choice(s). If meta-analysis was performed, describe the model(s), method(s) to identify the presence and extent of statistical heterogeneity, and software package(s) used.	p.7
	13e	Describe any methods used to explore possible causes of heterogeneity among study results (e.g. subgroup analysis, meta-regression).	p.7
	13f	Describe any sensitivity analyses conducted to assess robustness of the synthesized results.	Not performed
Reporting bias	14	Describe any methods used to assess risk of bias due to missing results in a synthesis (arising from reporting biases).	p.7 (funnel plot

Section and Topic	Item #	Checklist item	Location where item is reported
assessment			and Egger's test)
Certainty assessment	15	Describe any methods used to assess certainty (or confidence) in the body of evidence for an outcome.	Not performed
**RESULTS**			
Study selection	16a	Describe the results of the search and selection process, from the number of records identified in the search to the number of studies included in the review, ideally using a flow diagram.	8
	16b	Cite studies that might appear to meet the inclusion criteria, but which were excluded, and explain why they were excluded.	8
Study characteristics	17	Cite each included study and present its characteristics.	8, 9, 10 and Table 1.
Risk of bias in studies	18	Present assessments of risk of bias for each included study.	10 and Supplementary table 3
Results of individual studies	19	For all outcomes, present, for each study: (a) summary statistics for each group (where appropriate) and (b) an effect estimate and its precision (e.g. confidence/credible interval), ideally using structured tables or plots.	Table 2 and Table 3.
Results of syntheses	20a	For each synthesis, briefly summarise the characteristics and risk of bias among contributing studies.	Study characteristics (Table 1, p. 8–10); Risk of bias (Figure 2, Supplementary table 3, p.10).
	20b	Present results of all statistical syntheses conducted. If meta-analysis was done, present for each the summary estimate and its precision (e.g. confidence/credible interval) and measures of statistical heterogeneity. If comparing groups, describe the direction of the effect.	p. 11–14 and Figures 3–9
	20c	Present results of all investigations of possible causes of heterogeneity among study results.	p. 11–14 and Figures 3–9
	20d	Present results of all sensitivity analyses conducted to assess the robustness of the synthesized results.	Not performed
Reporting biases	21	Present assessments of risk of bias due to missing results (arising from reporting biases) for each synthesis assessed.	p. 11–14 and Figures 3–9
Certainty of evidence	22	Present assessments of certainty (or confidence) in the body of evidence for each outcome assessed.	Not performed
**DISCUSSION**			
Discussion	23a	Provide a general interpretation of the results in the context of other evidence.	p.14-18
	23b	Discuss any limitations of the evidence included in the review.	p.18,19,20
	23c	Discuss any limitations of the review processes used.	p.19
	23d	Discuss implications of the results for practice, policy, and future research.	p.20
**OTHER INFORMATION**			
Registration and	24a	Provide registration information for the review, including register name and registration number, or state that the review was not registered.	N/A

Section and Topic	Item #	Checklist item	Location where item is reported
protocol	24b	Indicate where the review protocol can be accessed, or state that a protocol was not prepared.	N/A
	24c	Describe and explain any amendments to information provided at registration or in the protocol.	N/A
Support	25	Describe sources of financial or non-financial support for the review, and the role of the funders or sponsors in the review.	p.22
Competing interests	26	Declare any competing interests of review authors.	p.22
Availability of data, code and other materials	27	Report which of the following are publicly available and where they can be found: template data collection forms; data extracted from included studies; data used for all analyses; analytic code; any other materials used in the review.	p.22

Supplementary table 2. Search strategy performed on Thursday, Nov[21st], 2024 using electronic databases

A. Search terms used in PubMed

Concepts	Sub-terms	Search options	Number of hits
**1. Sepsis**	1.1 sepsis	MeSH term	148,412 results
	1.2 sepsis	All fields	226,967 results
	1.3 septicemia	All fields	238,012 results
	1.4 septic shock	All fields	44,516 results
	1.5 systemic inflammatory response syndrome	All fields	162,760 results
	1.6 SIRS	All fields	7,325 results
	1.7 critical illness	MeSH term	41,766 results
	1.8 bloodstream infection	All fields	234,734 results
	Total 1= 1.1 OR 1.2 OR 1.3 OR 1.4 OR 1.5 OR 1.6 OR 1.7 OR 1.8		304,559 results
**2. Brachial FMD**	2.1 flow-mediated dilation	All fields	6,947 results
	2.2 flow-mediated vasodilation	All fields	4,912 results
	2.3 brachial FMD	All fields	3,720 results
	2.4 brachial artery	All fields	22,429 results
	2.5 brachial artery reactivity	All fields	2,498 results
	Total 2= 2.1 OR 2.2 OR 2.3 OR 2.4 OR 2.5		25,810 results
	Grand Total: 1 AND 2		205 results

B. Search terms used in Embase

Concepts	Sub-terms	Search options	Number of hits
**1. Sepsis**	1.1 sepsis	All fields	300,859 results
	1.2 septicemia	All fields	35,047 results
	1.3 septic shock	All fields	82,497 results
	1.4 systemic inflammatory response syndrome	All fields	29,549 results
	1.5 SIRS	All fields	16,322 results
	1.6 critical illness	All fields	49,458 results
	1.7 bloodstream infection	All fields	35,543 results
	Total 1= 1.1 OR 1.2 OR 1.3 OR 1.4 OR 1.5 OR 1.6 OR 1.7		466,615 results
**2. Brachial FMD**	2.1 flow-mediated dilation	All fields	8,173 results
	2.2 flow-mediated dilatation	All fields	5,165 results
	2.3 flow-mediated vasodilation	All fields	3,039 results
	2.4 brachial FMD	All fields	6,530 results
	2.5 brachial artery	All fields	39,803 results
	2.6 brachial artery reactivity	All fields	1,345 results
	Total 2= 2.1 OR 2.2 OR 2.3 OR 2.4 OR 2.5 OR 2.6		45,330 results
	Grand Total: 1 AND 2		404 results

C. Search terms used in Scopus

Concepts	Sub-terms	Search options	Number of hits
**1. Sepsis**	1.1 sepsis	ti,ab,kw	241,457 results
	1.2 septicemia	ti,ab,kw	48,348 results
	1.3 septic shock	ti,ab,kw	71,732 results
	1.4 systemic inflammatory response syndrome	ti,ab,kw	27,855 results
	1.5 SIRS	ti,ab,kw	66,404 results
	1.6 critical illness	ti,ab,kw	90,835 results
	1.7 bloodstream infection	ti,ab,kw	29,061 results
	Total 1= 1.1 OR 1.2 OR 1.3 OR 1.4 OR 1.5 OR 1.6 OR 1.7		493,831 results
**2. Brachial FMD**	2.1 flow-mediated dilation	ti,ab,kw	5,776 results
	2.2 flow-mediated dilatation	ti,ab,kw	3,852 results
	2.3 flow-mediated vasodilation	ti,ab,kw	4,484 results
	2.4 brachial FMD	ti,ab,kw	4,091 results
	2.5 brachial artery	ti,ab,kw	35,634 results
	2.6 brachial artery reactivity	ti,ab,kw	1,127 results
	Total 2= 2.1 OR 2.2 OR 2.3 OR 2.4 OR 2.5 OR 2.6		39,227 results
	Grand Total: 1 AND 2		690 results

D. Search terms used in Web of Sciences

Concepts	Sub-terms	Search options	Number of hits
**1. Sepsis**	1.1 sepsis	Abstract	99,534 results
	1.2 septicemia	Abstract	14,371 results
	1.3 septic shock	Abstract	23,243 results
	1.4 systemic inflammatory response syndrome	Abstract	8,357 results
	1.5 SIRS	Abstract	30,319 results
	1.6 critical illness	Abstract	22,708 results
	1.7 bloodstream infection	Abstract	16,014 results
	Total 1= 1.1 OR 1.2 OR 1.3 OR 1.4 OR 1.5 OR 1.6 OR 1.7		191,675 results
**2. Brachial FMD**	2.1 flow-mediated dilation	Abstract	4,281 results
	2.2 flow-mediated dilatation	Abstract	2,085 results
	2.3 flow-mediated vasodilation	Abstract	1,661 results
	2.4 brachial FMD	Abstract	3,236 results
	2.5 brachial artery	Abstract	14,714 results
	2.6 brachial artery reactivity	Abstract	476 results
	Total 2= 2.1 OR 2.2 OR 2.3 OR 2.4 OR 2.5 OR 2.6		18,033 results
	Grand Total: 1 AND 2		69 results

The total number of articles is 1,368

Supplementary Table 3. FMD ultrasound protocols in the included studies

Study (Year)	Patient position	Arm/hand position	Imaging site	Cuff location	Cuff pressure	Occlusion duration (min)	Rest / environment	Probe / mode	Post-deflation acquisition	Diameter measurement & analysis	Doppler usage	Operator / blinding
**Ravikumar et al., 2023**	Supine	Arm extended	~2 cm above antecubital fossa	Forearm, 1–2 cm distal to ACF	NR	5	NR	2D + pulse-wave Doppler	Doppler at 15 s; 2D at 30–90 s every ~15 s	Interval diameters; same investigator for all	Yes	Same investigator (inter-observer minimized)
**Fayed et al., 2021**	NR	NR	NR	NR	NR	NR	NR	NR	NR	NR	NR	NR
**Omar et al., 2020**	NR	Arm extended; skin marked	~2 cm above medial ACF	Forearm, 1–2 cm distal to ACF	200 mmHg (or +50 mmHg if SBP > 150)	5	NR (≥10-min of supine rest)	7–10 MHz; B-mode + pulse-wave	Doppler for 15 s after deflation	B-mode diameter; fixed probe/arm position<br>Baseline (≥10-min of supine rest & reactive hyperaemia	Yes	NR
**Junior et al., 2019**	NR	Arm abducted ~80°; forearm supinated	1–3 cm proximal to ACF (longitudinal; 60° insonation)	Forearm	200 mmHg	5	NR	10 MHz; pulsed Doppler	Continuous images for 3 min (RH)	Continuous image for 3 minutes<br>Baseline & post	Yes	NR
**Nelson et al., 2016**	Supine	NR	Probe proximal to cuff (brachial implied)	Upper arm near elbow (distal to probe)	Suprasystolic 250 mmHg	5	10-min rest	Doppler; occlusion verified continuously	Diameter & velocity continuously for 2 min	Continuous	Yes	NR
**Becker et al., 2012**	Supine	Arm fixed; no lines	Brachial (site	Forearm	230–250 mmHg	5	NR	7.5–10 MHz;	Diameter at 45–60 s	Diastolic diameter in 3	NR	NR

			rel. to ACF NR)					B-mode (IMT also)		adjacent segments × 3 beats; mean of 9 pre and 9 post		
**Wexler et al., 2012**	Supine, head ↑~30°, ≥10-min rest	Arm extended; thumb up	Medial approach, ~2 cm above ACF	Forearm, 1–2 cm distal to ACF	200 mmHg (or +50 mmHg if SBP > 150)	5	≥10-min rest	13-14 MHz; 2D (freq NR)	30–90 s at ~15-s intervals	End-diastolic diameter; 3 baseline & 3 post averages; 3 maximal post used	Yes	NR
**Vaudo et al., 2008**	Supine (non-dominant arm)	NR	Just above antecubital crease (longitudinal)	Forearm (most proximal portion)	230–250 mmHg	4	10–20 min rest; quiet, dark; 22 °C	5-12 MHz; B-mode; ECG-gated (R-wave)	Diameter at 45–60 s	Average of three basal and three post diameters	NR	One investigator; blinded to clinical data & sequence

Supplementary table 4. New-Castle-Ottawa scale (NOS) for quality assessment of studies

A. **Cohort studies**

	Ravikumar et al. 2023	Omar et al. 2020	Junior et al. 2019	Becker et al. 2012	Wexler et al. 2012	Vaudo et al. 2008
**Selection (maximum= 4)**						
Representativeness of the exposed cohort	★	★	★	★	★	-
Selection of the non-exposed cohort	★	-	N/A	★	★	★
Ascertainment of exposure	★	★	★	★	★	★
Demonstration that outcome of interest was not present at start of study	★	★	★	★	★	★
**Comparability (maximum= 2)**						
Comparability of cohorts on the basis of the design or analysis	★★	-	-	★	★★	★★
**Outcome (maximum= 3)**						
Assessment of outcome	★	★	★	★	★	★
Was follow-up long enough for outcomes to occur	★	★	★	★	★	★
Adequacy of follow up of cohorts	★	★	★	★	★	★
**Total Quality Score (maximum=9)**	9	6	6	8	9	8
**Quality Assessment**	Good	Moderate	Moderate	Good	Good	Good

A study can be awarded a maximum of one star (★) for each item in the selection and outcome category, and a maximum of two stars within comparability. A dash (–) indicates the study did not meet the criterion. N/A indicates the item was not applicable because the study was a single-arm cohort. A study was classified as high quality if the total score was (7–9 stars), moderate quality if (4–6 stars), and poor quality if (0–3 stars).

B. Case-control studies

	Fayed et al. 2021	Nelson et al. 2016
**Selection (maximum = 4)**		
Is the case definition adequate?	★	★
Representativeness of the cases	★	-
Selection of Controls	★	★
Definition of Controls	★	★
**Comparability (maximum= 2)**		
Comparability of cases and controls on the basis of the design or analysis	★	★
**Exposure (maximum= 3)**		
Ascertainment of exposure	N/A	N/A
Same method of ascertainment for cases and controls	N/A	N/A
Non-Response rate	-	-
**Total Quality Score (maximum= 9)**	5	4
**Quality Assessment**	Moderate	Moderate

A study can be awarded a maximum of one star (★) for each item in the selection and exposure category, and a maximum of two stars within comparability. A dash (–) indicates the study did not meet the criterion; N/A indicates the item was not applicable because sepsis status itself was treated as the “exposure”, which by definition differs between cases and controls. A study was classified as high quality if the total score was (7–9 stars), moderate quality if (4–6 stars), and poor quality if (0–3 stars).
